# High expression of IL-17 and IL-17RE associate with poor prognosis of hepatocellular carcinoma

**DOI:** 10.1186/1756-9966-32-3

**Published:** 2013-01-11

**Authors:** Rui Liao, Jian Sun, Han Wu, Yong Yi, Jia-Xing Wang, Hong-Wei He, Xiao-Yan Cai, Jian Zhou, Yun-Feng Cheng, Jia Fan, Shuang-Jian Qiu

**Affiliations:** 1 Liver Cancer Institute, Zhongshan Hospital, Fudan University, 136 Yi Xue Yuan Rd, Shanghai 200032, China; 2 Key Laboratory of Carcinogenesis and Cancer Invasion, the Chinese Ministry of Education, Shanghai, China; 3 Biomedical Research Center, Zhongshan Hospital, Fudan University, Shanghai, China

**Keywords:** Interleukin-17, Receptor, Hepatic stellate cell, Prognosis, Hepatocellular carcinoma

## Abstract

**Background:**

Hepatocellular carcinoma (HCC) is a typical malignancy in a background of chronic inflammation. Th17 cells (a major source of IL-17) constitute crucial components of infiltrating inflammatory/immune cells in HCC and can amplify inflammatory response via binding to interleukin-17 receptor (IL-17R). Thus, we investigated the expression and clinical significance of IL-17 and IL-17 receptor family cytokines in HCC.

**Methods:**

The expression and prognostic value of IL-17 and IL-17R (A-E) were examined in 300 HCC patients after resection. Six Th17 associated cytokines in serum (n = 111) were quantified using enzyme-linked immunosorbent assays. Phenotypic features of IL-17^+^ CD4^+^ T cells were determined by flow cytometry analysis.

**Results:**

High expression of intratumoral IL-17 and IL1-7RE were significantly associated with poorer survival (p = 0.016 and <0.001, respectively) and increased recurrence (both P < 0.001) of HCC patients. Moreover, intratumoral IL-17, individually or synergistically with IL-17RE, could predict HCC early recurrence and late recurrence. Also, peritumoral IL-17RE showed the prognostic ability in HCC (P < 0.001 for OS/TTR). Furthermore, expression levels of Th17 associated cytokines including IL-6, -22, -17R and TNF-α were increased in serum of HCC patients compared to haemangioma patients. Importantly, activated human hepatic stellate cells induced in vitro expansion of IL-17^+^ CD4^+^ T cells.

**Conclusions:**

High expression of IL-17 and IL-17RE were promising predictors for poor outcome of HCC patients. The protumor power of IL-17 producing CD4^+^ T cells was probably involved in the crosstalk with different types of inflammatory/immune cells in HCC.

## Background

Hepatocellular carcinoma (HCC) is a typical malignancy that slowly unfolds on a background of chronic inflammation mainly due to exposure to hepatitis viral infection and cirrhosis [1]. Thus, to a large extent, HCC metastatic biologic behavior and poor prognosis may be determined and/or influenced by the local inflammatory status [2]. We have previously demonstrated that the densities of tumor-associated macrophages [3], neutrophils [4] and regulatory T cells [5] were selectively associated with poor prognosis of HCC patients. Moreover, some inflammatory/immune cells may cooperate with each other to acquire more potent tumor-promoting activities and result in poorer prognosis, such as combination of peritumoral mast cells and T-regulatory cells [6]. Notably, some inflammatory cytokines expression levels like interleukin-2, -15 [7] and −17 [8], predominantly produced by Th1, Th2 and Th17, are associated with HCC recurrence and survival. These results supported that “context” of inflammation had a potential shift from pro-inflammatory response toward tumor-promoting direction.

A subset of IL-17 producing CD4^+^ T cells (Th17), preferentially producing IL-17A, IL-17F and IL-22 [8,9], have been recently appreciated as important regulators in human tumors [10]. However, the protumoral or antitumoral activity of Th17 cells remained controversial [11,12]. Indeed, collective evidence suggested that the confusing Th17 cells function in tumor arose from the effect of IL-17 itself, which may depend on different tumor microenvironments in various tumor type, location and stage of disease [12,13]. In HCC, increased IL-17-producing cell infiltrations have been demonstrated to correlate with poor prognosis [8]. A series of data indicated IL-17 could promote tumor progression through neutrophil recruitment [14,15] and targeting tumor cells directly to activate some signaling pathways such as AKT [14] and NF-κB [16]. A recent study [17] revealed that Th17 cells were implicated in a fine-tuned collaborative action with activated monocytes toward a tumor-promoting direction in HCC. Considering IL-17 receptor (IL-17R) is expressed ubiquitously on all types of liver cells [18], IL-17 producing cells were most likely involved in the crosstalk with various liver-resident cells in HCC. Interestingly, our conjecture was partly supported by a report that IL-17 producing cells could process in a paracrine manner by surrounding IL-17 receptor-positive cells such as hepatic stellate cells (HSCs) [19]. But so far, only limited attention has been paid on the effects of resident inflammatory/immune cells on IL-17 producing cells in HCC. Also, clinical relationships of IL-17 and IL-17 receptor family cytokines in HCC are still unknown.

In this study, we demonstrated high expression of IL-17 and IL-17RE were promising predictors for poor outcome of HCC after resection, and activated human HSCs induced in vitro expansion of IL-17 producing CD4^+^ T cells, therefore indicating the intrinsic association among various inflammatory/immune cells and cytokines involved in the progress of tumor.

## Materials and methods

### Patients and specimens

All archival specimens were obtained from 300 consecutive HCC patients after surgical resection in 2007 (Table 1). A total of 111 serum samples of preoperative and postoperative (at 5 days) HCC and preoperative haemangioma patients were prospectively collected at our hospital from January to July in 2011. Haemangioma patients had normal liver function in this cohort relative to normal, age matched donors. The experimental protocols described in this study complied with the Ethics Review Committee of Zhongshan Hospital of Fudan University, and every patient provided written informed consent before enrollment.

**Table 1:** Peritumoral and intratumoral IL-17RE expression according to characteristics of 300 HCC patients

**Characteristics**		**Peritumoral IL-17RE**			**Intratumoral IL-17RE**		
		**Low**	**high**	**p**	**Low**	**high**	**p**
		**n = 176**	**n = 124**		**n = 221**	**n = 79**	
Gender	Male	144	109	0.197	187	66	0.857
	Female	32	15	34	13		
Age(years)	≤53	90	67	0.640	121	36	0.190
	>53	86	57	100	43		
ALT(U/L)	≤75	154	109	1.000	193	70	0.844
	>75	22	15	28	9		
AFP(ng/ml)	>20	104	87	0.52	138	53	0.498
	≤20	72	37		83	26	
Hepatitis history	Yes	130	88	0.601	62	20	0.769
	No	46	36	159	59		
Cirrhosis	Yes	155	110	1.000	199	66	0.152
	No	21	14	22	13		
Vascular invasion	Yes	38	46	0.004	61	23	0.884
	No	138	78	160	56		
Encapsulation	Yes	89	68	0.483	114	43	0.695
	No	87	56	107	36		
Number	Single	155	108	0.859	196	67	0.425
	Multiple	21	16	25	12		
Size(cm)	≤5	122	72	0.50	145	49	0.585
	>5	54	52	76	30		
Differentiation	I-II	128	92	0.793	166	54	0.299
	III-IV	48	32	55	25		
TNM stage	I	129	73	0.012	150	52	0.780
	II-III	47	51	71	27		

IL-17RE: interleukin-17receptor E; AFP: alpha fetoprotein; ALT, alanine aminotransferase; TNM, tumor-node-metastasis.

### Tissue microarray design and immunocytochemistry

TMAs were constructed as described previously [20]. All patients were monitored postoperatively until January 2012. The total numbers of positive cells of each core were evaluated by two independent investigators blind to clinical outcome and knowledge of the clinicopathologic data. Positive staining cells were screened (100X) and four most representative areas were observed (400X) to count using a Leica DMLA light microscope (Leica Microsystems, Wetzlar, Germany). Data were expressed as the mean (±SE) number cells for one computerized 400X microscopic field based on the triplicate samples obtained from each patient.

Immunohistochemistry of paraffin sections was carried out using streptavidin peroxidase conjugated method as described previously [20]. Briefly, primary antibodies were added on the slides to incubate at 4°C overnight. After washing with phosphate buffered solution (PBS) for three times, secondary antibodies were incubated at 37°C for 1 hour. Following incubation with streptoavidin-labeled horseradish peroxidase at room temperature for 30 minutes, tissues were stained with DAB chromogenic agent under light microscope. Antibodies of IL-17 and IL-17R (A-E) were used (R&D Systems and Sigma-Aldrich, dilution from 1:50–200).

### Enzyme-linked immunosorbent assay (ELISA) in serum

IL-6, -9, -17, -22, -17R and TNF-α levels in serum were determined using ELISA kits (IL-6, -17 and TNF-α, R&D Systems; IL-9 and 22, eBioscience; IL-17R, RayBio) according to the manufacturers’ instructions.

### Isolation and culture of cells

As described previously [21], peripheral blood mononuclear cells were isolated from the blood of 12 HCC patients and 10 haemangioma patients by LymphoPrep™ (Axis-Shield) gradient centrifugation as described previously [21], and cultured in RPMI1640 containing 10% fetal calf serum and 1% penicillin/streptomycin.

Activated human hepatic stellate cells (HSCs) were isolated from peritumoral hepatic tissues at distances of 1 cm from the tumor margin as our described previously [20] and cultured in Dulbecco’s modified Eagle medium (DMEM) containing 10% fetal calf serum and 1% penicillin/streptomycin. Briefly, after combined digestion of liver tissue with pronase, collagenase and DNase, HSCs were separated from other nonparenchymal cells by centrifugation over a gradient of 11% Nycodenz (Axis-shield) at 1400g for 20 minutes. Average yield per isolation were 1 × 10^7^ HSCs/20g liver. HSCs purity was assessed by the autofluorescence property and morphology, the populations were more than 90% pure and 95% viable. After passage, activated HSCs purity was 100%, assessed by α-SMA staining. Activated HSCs were studied between serial passages 3 and 6.

### Preparation of conditioned medium (CM) and flow cytometry analysis

Conditioned medium (CM) of HSCs was collected as described previously [20]. Briefly, after seeding into T25 flasks (0.6×10^6^ cells/5ml) for 24 hours, HSCs were washed twice with serum-free RPMI1640, and then incubated for another 24 hours with serum-free RPMI1640.CM was then collected, centrifuged to remove cell debris, filtered, and stored at −20°C until use.

5×10^5^ peripheral lymphocytes were cultured in a 24-well plate and resuspended in a 1:1 mixture of fresh CM of HSCs or control medium (RPMI1640 with 5%FBS). After a proliferation time of 7 days with CM of HSCs or control medium, and IL-6 and TGF-β stimulation in the presence of 2 mg/ml anti-CD3 and 1 mg/ml anti-CD28 [22,23], cells were washed twice with PBS. Then, the peripheral lymphocytes were detected followed by 5 hours stimulation with 50 ng/ml phorbol 12-myristate 13-acetate (PMA, Sigma) and 500 ng/ml Ionomycin (Sigma) in the presence of 0.7 ul/ml GolgiStop™ (BD Biosciences). Thereafter, cells were stained with surface markers, fixed and permeabilized, and stained with intracellular marker. Finally, cells were fixed with 4% paraformaldehyde for flow cytometry analysis. The fluorochrome-conjugated antibodies used (FITC-conjugated CD4, BD Pharmingen; PE-conjugated CD3 and APC-conjugated IL-17A from eBioscience).

### Statistic analysis

Statistical analysis was completed with SPSS 16.0 (SPSS, Inc., Chicago, IL) and *P <* 0.05 was considered statistically significant. The Student *t* test, Fisher’s exact tests, *χ*^2^ tests and Spearman ρ coefficients tests were used as appropriate for the comparison of variables. Univariate analysis and multivariate Cox proportional hazards model was performed to estimate independent prognostic factors. The “minimum *p* value” approach [4] was used to get an optimal cut-off by X-tile 3.6.1 software (Yale University, New Haven, CT, USA).

## Results

### Immunohistochemical characteristics of IL-17 receptor family members in HCC

**Figure 1:**
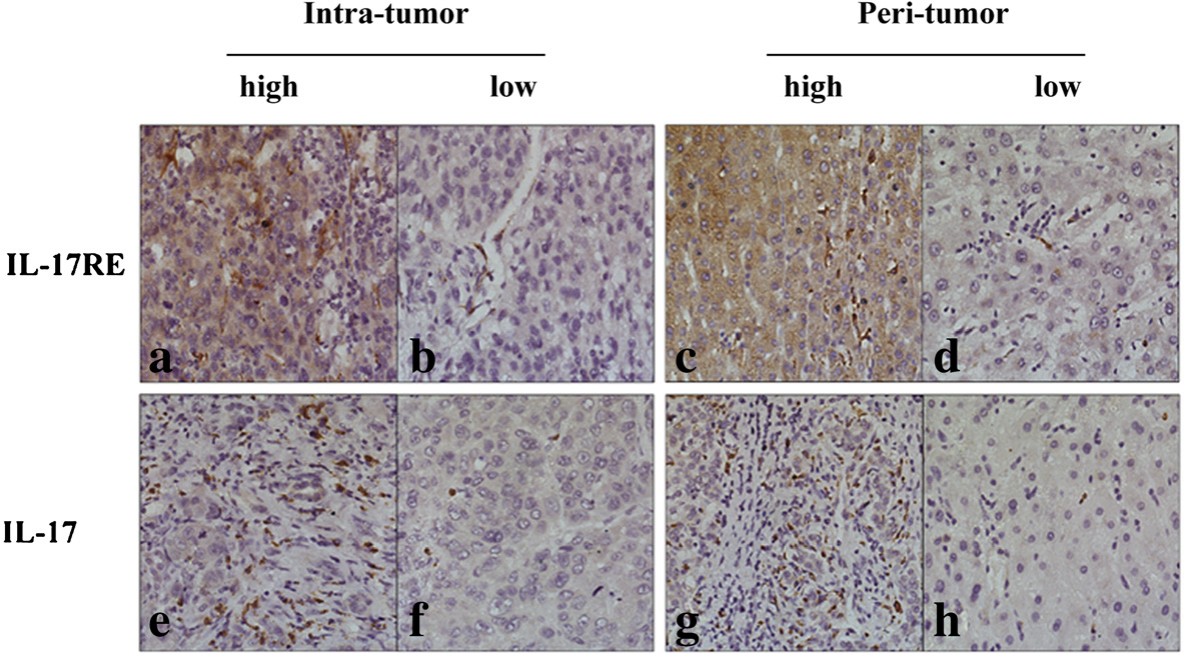
**Immunohistochemistry analysis of IL-17RE and IL-17. a-h **showed high (**a**, **c**, **e **and **g**) and low (**b**, **d**, **f** and **h**) densities of IL-17RE and IL-17 staining cells in intratumoral (**a**, **b**, **e **and **f**) and peritumoral area (**c**, **d**, **g **and **h**), respectively (x 200).

As shown in Figure 1 and Additional file 1, IL-17 receptor family members were focal, scattered and diffuse on various liver cells and cancer cells, which showed membrane or cytoplasm staining and a variety of staining patterns, including different positive cells rates and staining intensity. The localization of IL-17RA was very similar to that of IL-17RB. The expression patterns of them in tissues were diffuse, and most of them showed strong positive expression levels (peritumoral IL-17RA and IL-17RB: 177/300 and 209/300; intratumoral IL-17RA and IL-17RB: 186/300 and 209/300, respectively) according to positive cells population and magnitude of staining [21]. In contrast to IL-17RA, IL-17RC expression was much weaker in both peritumoral and intratumoral tissues, although it was identified as a receptor of IL-17, pairing with IL-17RA to induce responses to IL-17 [24]. Moreover, IL-17RD and IL-17RE were located in similar staining patterns in stromal cells besides parenchymal cells.

### Identification of prognostic cytokines from IL-17 receptor family members and IL-17

**Figure 2:**
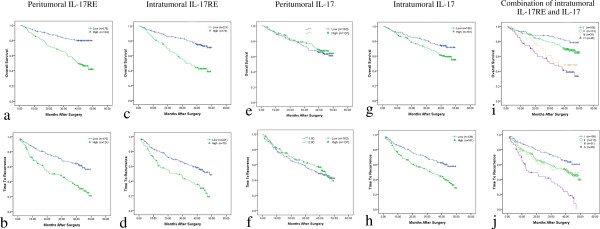
**Prognostic significance of peritumoral IL-17RE, intratumoral IL-17RE and IL-17. **High density of peritumoral IL-17RE (**a **and **b**), intratumoral IL-17RE (**c **and **d**) and intratumoral IL-17 (**g** and **h**) were related to decreased overall survival (OS, **a**, **c** and **g**) and time to recurrence (TTR, **b**, **d **and **h**). Combination of intratumoral IL-17RE and IL-17 was also associated with OS (**i**) and TTR (**j**). I: both low density; II: low IL-17RE but high IL-17 density; III: high IL-17RE but low IL-17 density; and IV: both high density. Peritumoral IL-17 (**e **and **f**) showed no predictive value for OS (**e**) and TTR (**f**).

The “minimum p value” approach [4] was used to get an optimal cut-off (intratumoral IL-17RE and IL-17, and peritumoralIL-17RE were 71, 51 and 48, respectively) for the best separation of patients related to time to recurrence (TTR) or overall survival (OS). Firstly, we analyzed the potential prognostic value from 5 IL-17 receptor family members. Of the 5 receptors tested in this study, IL-17RE density was significantly associated with TTR and OR in both peritumoral and intratumoral tissues (all P < 0.001, Table 2). Other four receptors were found no significant relationship with prognosis of these HCC patients. Then, prognostic influence of IL-17 was evaluated to further investigate the possible association between the related parameters. We found that intratumoral IL-17 density was an independent prognostic factor in this HCC cohort (Table 2). Furthermore, the prognostic ability of the combination of intratumoral IL-17RE and IL-17 densities was revalued. Patients were classified into four groups (Figure 2): I: both low density (n = 108); II: low IL-17RE but high IL-17 density (n = 113); III: high IL-17RE but low IL-17 density (n = 31); and IV: both high density (n = 48). Significant discrepancy in OS (P <0.001) and TTR (P < 0.001) were found (both low vs both high, Table 2 and Figure 2).

**Table 2:** Prognostic factors for survival and recurrence

**Factor**	**OS**			**TTR**		
	**Univeriate**	**Multivariate**		**Univeriate**	**Multivariate**	
	***P***	**HR (95% CI)**	***P***	***P***	**HR (95% CI)**	***P***
AFP(ng/ml) (≤20 v >20)	0.022		NS	0.003	1.482(1.030-2.132)	0.034
Tumor number (single v multiple)	<0.001	2.803(1.616-4.864)	<0.001	0.011	1.964(1.395-2.766)	0.001
Vascular invasion (yes v no)	<0.001	1.571(1.027-2.401)	0.037	<0.001		NS
Tumor size(cm) (≤5.0 v >5.0)	<0.001	2.552(1.671-3.897)	<0.001	<0.001	1.964(1.395-2.766)	<0.001
TNM stage (I v II- III)	<0.001	1.891(1.223-2.926)	0.004	0.001	1.564(1.092-2.240)	0.015
Peritumoral density (low v high) IL-17RE	<0.001	2.172(1.404-3.361)	<0.001	<0.001	1.721(1.222-2.425)	0.002
Intratumoral density (low v high)						
IL-17RE	<0.001		NS	<0.001		NS
Il-17	0.016		NS	<0.001		NS
Combination of IL-17RE &IL-17	<0.001	1.569(1.315-1.873)	<0.001	<0.001	1.433(1.234-1.663)	<0.001

Univeriate analysis: Kaplan-Meier method; multivariate analysis: Cox proportional hazards regression model.

Abbreviations: *OS*, overall survival; *TTR*, time to recurrence; *HR*, Hazard Ratio; *CI*, confidence interval; *AFP*, alpha fetoprotein; *TNM*, tumor-node-metastasis;*IL-17RE*, interleukin-17receptor E; N*A*, not adopted; *NS*, not significant.

### Association of IL-17RE/IL-17 with clinicopathologic variables and univariate and multivariate analyses of the prognostic abilities

**Figure 3:**
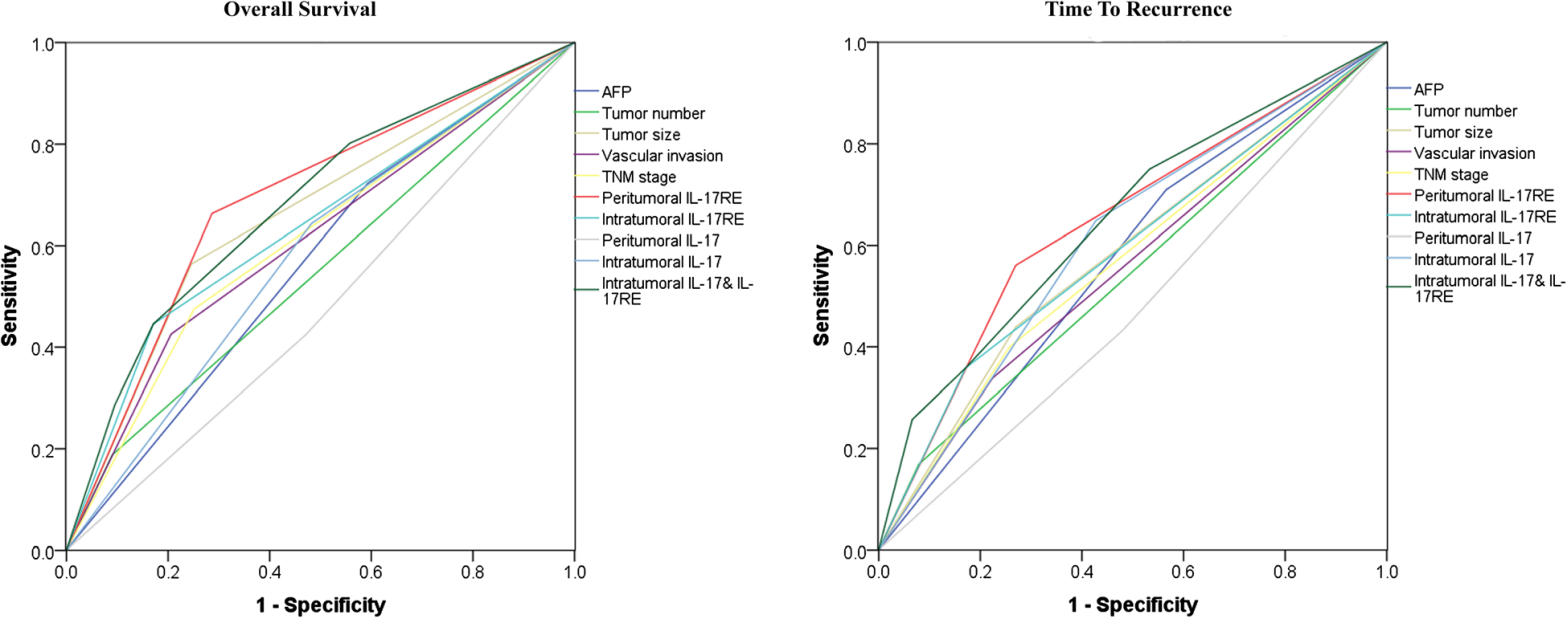
**Receiver operating characteristic analysis based on (a) overall survival and (b) time to recurrence of 300 HCC patients. **Peritumoral IL-17RE expression (AUC_TTR_ = 0.646, P < 0.001; AUC_OS_ = 0.688, p < 0.001), intratumoral IL-17 expression (AUC_TTR_ = 0.611, P < 0.001; AUC_OS_ = 0.581, p = 0.023), peritumoral IL-17 expression (AUC_TTR_ = 0.476, P = 0.474; AUC_OS_ = 0.477, p = 0.509), intratumoral IL-17RE expression (AUC_TTR_ = 0.646, P = 0.005; AUC_OS_ = 0.637, p < 0.001), combination of intratumoral IL-17 and IL-17RE expression (AUC_TTR_ = 0.650, P <0.001; AUC_OS_ = 0.681, p < 0.001), AFP (AUC_TTR_ = 0.572, P =0.031; AUC_OS_ = 0.565, p = 0.066), tumor number (AUC_TTR_ = 0.545, P =0.178; AUC_OS_ = 0.549, p = 0.167), vascular invasion (AUC_TTR_ = 0.557, P =0.087; AUC_OS_ = 0.610, p = 0.002), tumor size (AUC_TTR_ = 0.585, P =0.011; AUC_OS_ = 0.659, p < 0.001), TNM stage (AUC_TTR_ = 0.571, P =0.033; AUC_OS_ = 0.612, p = 0.002).

In this whole study population, the 1-, 3- and 5-year OS and RFS rates were 88.9%, 70.9%, 61.6%, and 78.2%, 55.9% and 38.6%, respectively. As shown in Table 1, none of clinicopathologic variables was found to be associated with expression levels of intratumoral IL-17RE and IL-17. In contrast, peritumoral IL-17RE density had relationship with vascular invasion (P = 0.004) and late TNM stage (P = 0.012). On univariate analysis of our data, several clinical factors including AFP, tumor multiplicity, tumor size, vascular invasion and TNM stage showed prognostic significance for both OS and TTR (Table 2). Then, significant clinical factors were used for further multivariate analysis. Tumor number, tumor size and TNM stage were demonstrated to be related with OS (P < 0.001, <0.001 and =0.004) and TTR (P = 0.001, <0.001 and =0.015), respectively. While vascular invasion was an independent predictor for OS (P = 0.037). Furthermore, combination of intratumoral IL-17RE and IL-17 densities showed higher predictive value on outcome of HCC patients by multivariate (Table 2) and predictive accuracy by ROC analysis (Figure 3) than either factor alone. To analyze the prognostic capacity of these biomarkers for early recurrence (metastasis after surgery ≤24 months) and late recurrence (new primary lesion after surgery >24 months) [4], Kaplan-Meier method was performed. Combination of intratumoral IL-17RE and IL-17 densities were found to be more likely to suffer from tumor early and late recurrences by univariate and multivariate analysis (Table 3). In addition, peritumoral IL-17RE density also showed the predictive power in OS and TTR (Figure 2).

**Table 3:** Prognostic factors for early and late recurrence

**Factor**	**Early recurrence**			**Late recurrence**		
	**Univeriate**	**Multivariate**		**Univeriate**	**Multivariate**	
	***P***	**HR (95% CI)**	***P***	***P***	**HR (95% CI)**	***P***
AFP(ng/ml)(≤20 v >20)	0.018	1.457(1.012-2.098)	0.043	NS		NA
Tumor size(cm) (≤5.0 v >5.0)	<0.001	1.799(1.272-2.544)	0.001	NS		NA
Vascular invasion(yes v no)	<0.001	1.472(1.032-2.101)	0.033	NS		NA
TNM stage (I v II- III)	0.001	1.423(1.003-2.020)	0.048	NS		NA
Peritumoral density (low v high) IL-17RE	<0.001	1.604(1.129-2.280)	0.008	0.001	2.148(1.158-3.986)	0.015
Intratumoral density (low v high)						
IL-17RE	0.001		NS	0.007		NS
Il-17	0.004		NS	0.034		NS
Combination of IL-17RE &IL-17	<0.001	1.430(1.227-1.666)	<0.001	<0.001	1.458(1.093-1.947)	0.010

Univeriate analysis: Kaplan-Meier method; multivariate analysis: Cox proportional hazards regression model.

Abbreviations: *HR*, Hazard Ratio; *CI*, confidence interval; *AFP*, alpha fetoprotein; *TNM*, tumor-node-metastasis;*IL-17(RE)*, interleukin-17(receptor E); *NA*, not adopted; *NS*, not significant.

### Expression levels of IL-6, -22, -17R and TNF-α were increased in serum of patients with HCC

**Figure 4:**
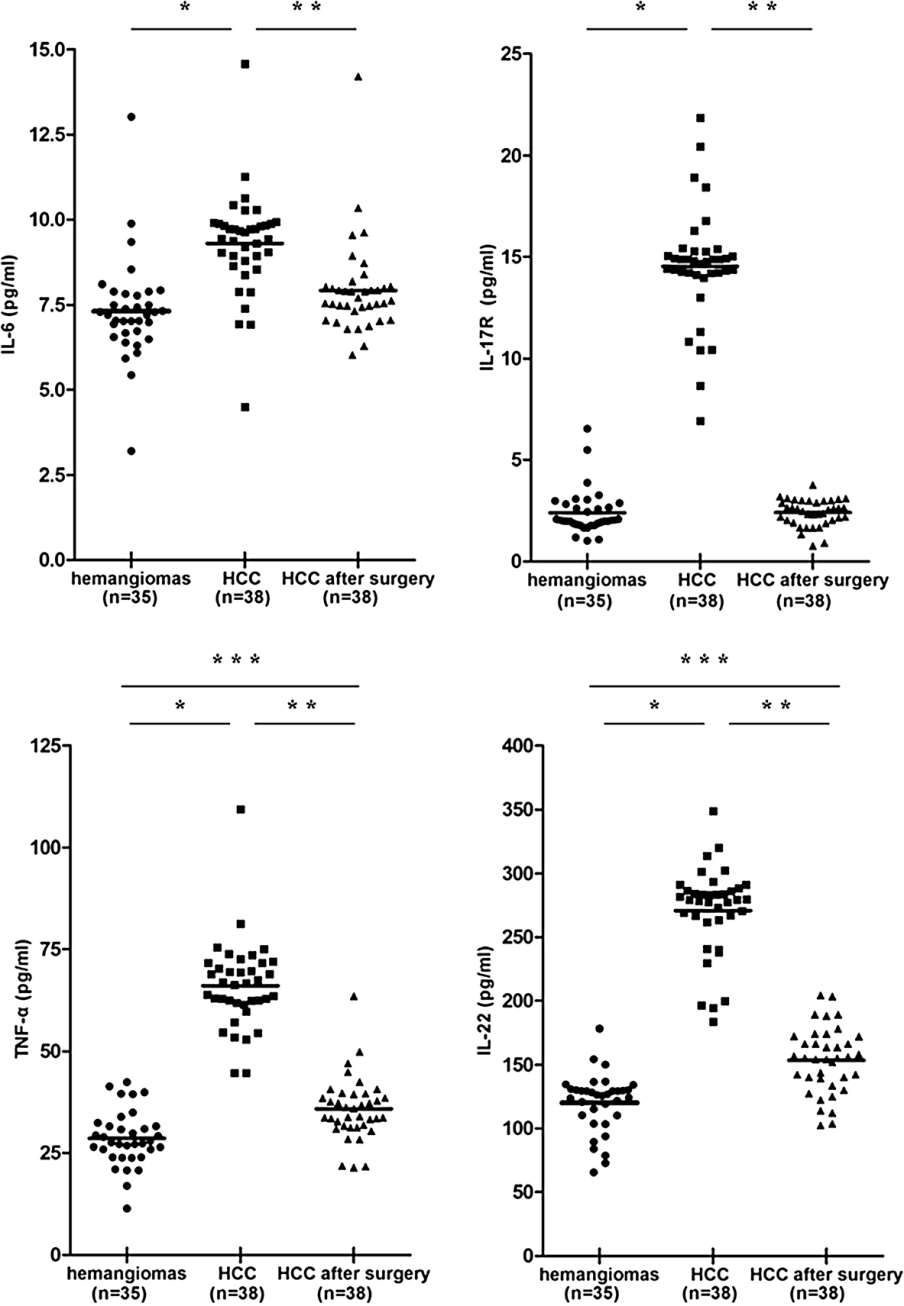
Increased expression levels of IL-6 (a), -22 (d), -17R (b) and tumor necrosis factor (TNF)-α (c) in serum of HCC patients. * P < 0.05, versus haemangioma patients; ** P < 0.05, versus postoperative patients; *** P < 0.05, versus haemangioma patients.

Among six investigated cytokines, the expression levels of IL-6 (9.30 ± 1.51 vs 7.32 ± 1.49pg/ml), -22 (270.83 ± 34.73 vs 120.19 ± 23.03pg/ml), -17R (14.52 ± 2.79 vs 2.40 ± 1.10pg/ml) and TNF-α (66.00 ± 10.85 vs 28.60 ± 6.80pg/ml) were significantly higher in HCC patients than hemangiomas patients (P < 0.001, Figure 4). At postoperative 5 days, all of their expression levels were decreased (P < 0.001). There was no difference for IL-9 (1.62 ± 0.50 vs 1.41 ± 0.62pg/ml) and IL-17 (5.24 ± 1.37 vs 5.33 ± 1.82pg/ml) between the groups of patients with HCC and hemangiomas (P > 0.05).

### Conditioned medium of peritumoral activated human HSCs induced expansion of circulating of IL-17 producing CD4^+^ T cells

**Figure 5:**
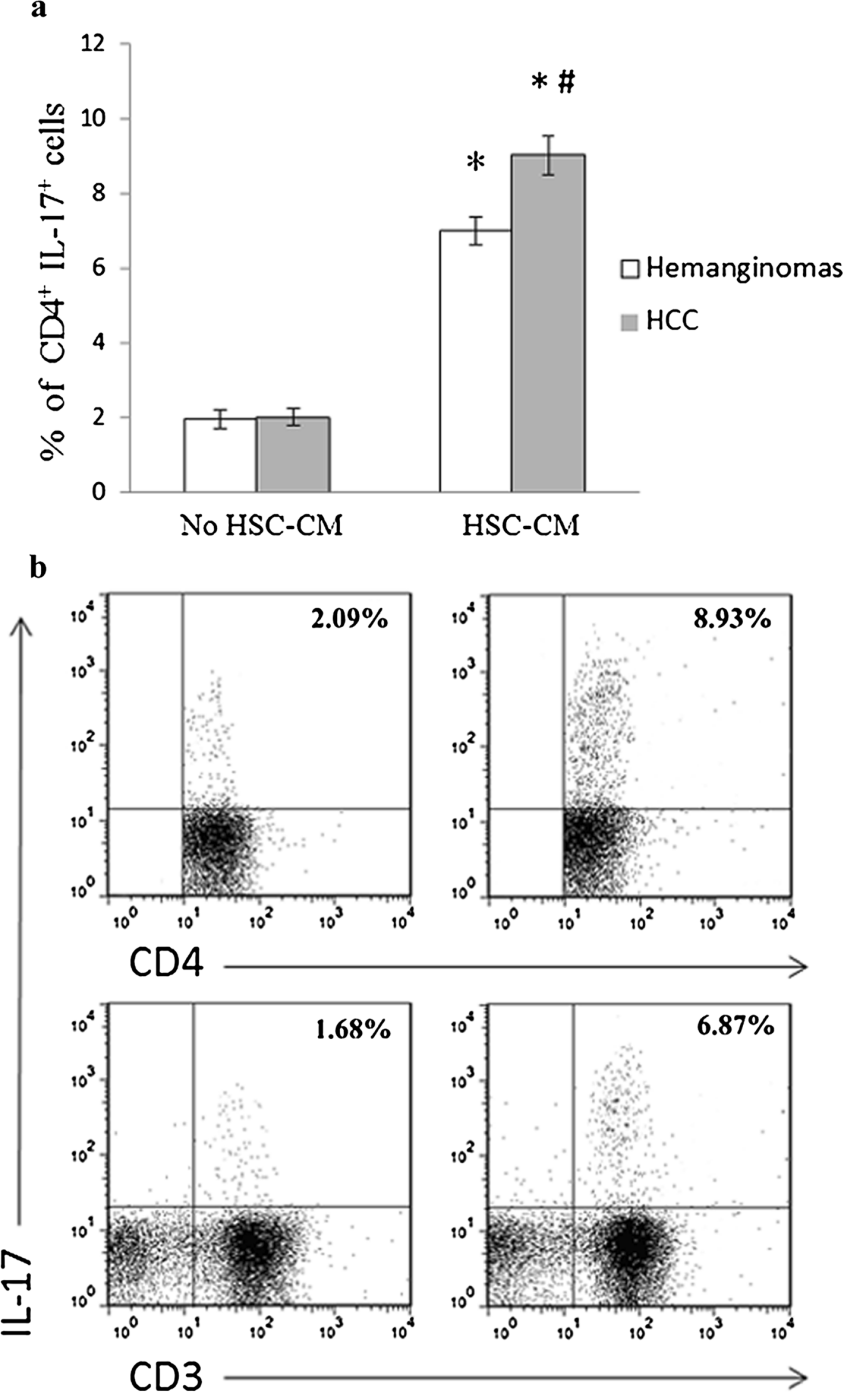
**Expansion of circulating of IL-17-producing CD4**^**+ **^**T cells induced by activated human hepatic stellate cells in vitro. a**: increased expression of circulating IL-17 producing CD4^+^ T cells in HCC patients after stimulation with conditioned medium (CM) which was determined by flow cytometry; **b**: the representative flow cytometry data from 12 HCC patients. The right panel was treated by a 1:1 mixture of fresh CM of HSCs or control medium (RPMI1640 with 5%FBS), and the left panel was only stimulated with control medium. *P <0.01 compared with IL-17-producing CD4^+ ^T cells before stimulation with CM; ^#^P <0.01 compared with haemangioma patients.

Human HSCs can express IL-17R [19] and modulate T-lymphocyte proliferation [25]. Here, we found that CM of human activated HSCs was related with in vitro proliferation of IL-17 CD4^+^ T cells (Figure 5 and Additional file 2). Notably, the frequency of IL-17^+^ CD4^+^ cells exposed to CM was increased both in HCC patients (from 2.03 ± 0.23% to 9.04 ± 0.52%, P < 0.01) and in hemangiomas patients (from 1.96 ± 0.25% to 7.02 ± 0.37%, P < 0.01). Consistently, IL17^+^ CD3^+^ T cells were also increased significantly after 7-days stimulation (P < 0.01). As shown in Figure 5a, there was no difference of primary peripheral CD4^+^ and CD3^+^ IL-17^+^ T cells without stimulation between the groups of HCC patients and hemangiomas patients (P > 0.05).

## Discussion

Recent attention has been paid to the prognostic ability and underlying molecular mechanisms of IL-17 producing cells to foster growth and progression of HCC [8,14]. However, research defining the relationships of IL-17 receptor family members and HCC has lagged. In the present study, we observed various expression patterns of IL-17 receptor family members in HCC tissues using immunohistochemistry, which probably suggested their distinct biological effects on tumor growth. Among these receptors, expression levels of IL-17RE exhibited specificity in prognostic ability for dismal outcome of patients with HCC. Compared to low subgroup, patients with high-density of IL-17RE have shorter OS and TTR in both intratumoral and peritumoral tissues. Therefore, patients with high density of IL-17RE need a close monitoring. IL-17RE may provide us a novel prognosticator for poor outcome of HCC patients after surgery.

High expression of intratumoral IL-17 was also related to the prognosis of HCC patients in this cohort, which drove us to investigate its correlation with IL-17RE. Combination of intratumoral IL-17RE and IL-17 densities yielded better predictive performance than them alone. These findings indicated intratumoral IL-17RE and IL-17 may be involved in a fine-tuned collaborative action in the procession of HCC. Although IL-17RE is the least well characterized cytokine of the IL-17 receptor family cytokines, a recent study [26] reported that IL-17RE could form heterodimeric complex with IL-17RA participating in induction of proinflammatory cytokines and chemokines. We therefore assumed that intratumoral IL17RE had a high degree of functional overlap with IL-17 producing cells and was responsible for aggressiveness of HCC cells, at least in form of heterodimeric complex with IL-17RA. Importantly, we documented that combination of intratumoral IL-17 and IL-17RE densities were associated with HCC recurrences which can be divided into early recurrence (≤24 months), a true metastasis caused by dissemination of cancer cells, and late recurrence (>24 months) originating from de novo hepatocarcinogenesis [4]. In this study, we proposed that IL-17 and IL-17RE orchestrated the protumor activities in the procession of HCC recurrence and progression due to the residual intrahepatic metastases as well as de novo cancer in the liver remnant.

In addition to the local immune response in liver tissue, expression levels of considerable soluble factors in circulation may reflect the systemic immune status of individuals with tumor and act as noninvasive markers for HCC screening and recurrence monitoring [27]. So, we evaluated the serum levels of Th17 associated cytokines/inflammatory mediators and found higher levels of IL-6, -17RA, -22 and TNF-α in HCC than those in haemangioma, suggesting their potential value as monitoring indictors in HCC. During inflammatory response, TNF-α and IL-17 can act in a synergistic manner to sustain neutrophil recruitment [28]. Recent evidence [10] found that IL-17 could enhance IL-6 production and subsequently promote tumor growth. On the other hand, IL-6 and IL-9 were critical initiators of Th17 differentiation and expansion which facilitate IL-17 secretion [29,30]. Interestingly, IL-22 has already been identified as a coexpression cytokine with IL-17 in Th17 cells and cooperatively induced an innate immune response [31]. Thus, we proposed that distinct expression levels of these cytokines may reflect their potential immune regulatory properties and synergistic interactions of cytokine networks in part via IL-17 signaling pathway. Moreover, the kinetics of cytokine products may serve as critical homeostatic factors in inflammatory “context” to determine the direction of tumor progression to some extent. In the present study, IL-17 expression level was not increased significantly. We speculated that compared with the circulating factors, fertile liver tissues (soil) endowed with abundant activated inflammatory/immune cells may play a more important role to determine IL-17 as a protumoral component. Obviously, numerous cytokines or growth factors involved in IL-17 pathway also need to be investigated such as IL-1, IL-23, TGF-β. In the absence of commercial human IL-17RE ELISA kit, we did not detect its expression in serum. Further study is required in our future research.

Despite several substantive studies [10,17] have confirmed the crosstalk with several types of inflammatory/immune cells contributed to the protumor power of Th17 (a major source of IL-17), knowledge of their interaction in HCC is still incomplete. In a recent study [20], we demonstrated HSCs were the vital inflammatory cells involved in the recurrence of HCC and could produce cytokines (IL-6 and TNF-α) to create a cytokine milieu that benefited the expansion of human Th17 cells [17]. Moreover, our recent gene expression profile of HSCs confirmed several IL-17 receptors (e.g. IL-17RA, RB and RE) were expressed in HSCs (data not shown). Inasmuch as the function of HSCs as liver-resident antigen-presenting cells [32], we identified the phenotypic features of IL-17 producing CD4^+^ T cells with the influence of HSCs in vitro. Interestingly, our present investigation provided evidence that secretions of activated human HSCs induced in vitro expansion of IL-17^+^ CD4^+^ T cells in HCC. In contrast, a recent data indicated suppressing Th17 differentiation by mouse HSCs [23]. Several aspects may contribute to this discrepancy. The first could be the different species (human *vs* mouse). Second, we used conditioned medium of HSCs, not per se HSCs, in order to eliminate the effects of other T cells on HSCs and subsequently feedback responses. Third, activation of HSCs can led to the loss of retinoic acid (RA) [33] which has already been identified as a key regulator to inhibit the generation of Th17 [34]. Therefore, absence of RA and in vitro activation made human HSCs appear to be fibroblast-like cells which were addressed to promote the expansion of Th17 [35]. Most recently, we found TREM-1 was a pro-tumor gene in HSCs [20], however, two independent studies on human gene expression profile displayed its contradictory roles in development of T cells: enhancing Th1 priming in mature dendritic cells [36] and dampening Th1 and Th17 responses in monocytes [37]. Therefore, the same gene in different cells appears to bias certain function toward an alternatively activated phenotype, suggesting the mechanistic complexity in signal integration of functional genes in various cells. A detailed understanding needs to be investigated.

In this study, we only studied some representative inflammatory mediators and the blood sample size was not large. Additionally, response to the stimulation of activated HSCs, the roles of memory and naïve CD4^+^ T cells in expansion of IL-17^+^ cells should be different. Various synergistic effects from other T cells or secretions in PBMC may participate in this process. We believe there are more linkages between activated HSCs, IL-17 and their receptors than what involved in this study. Therefore, extensive studies are needed in the future.

## Conclusions

In conclusion, we have shown that the high expression of IL-17 and IL-17RE in HCC were associated with worse clinical outcome after resection. The protumor power of IL-17 producing CD4^+^ T cells was probably involved in the mechanisms of inflammatory response interacting with different types of inflammatory/immune cells in HCC. In this regard, IL-17 and IL-17RE, acting as tumor promoters, may provide useful predictors for triaging at-risk patients with recurrence and metastasis of HCC following resection and also possible therapeutic targets against this disease.

## Competing interests

The authors declare that they have no competing interests.

## Authors’ contributions

RL and JS conceived and designed the experiments. HW, YY, JXW and HWH contributed to the acquisition of the data, XYC has made substantial contribution to collected tissue samples, JZ, YFC, JF and SJ Q participated in study design and coordination, data analysis and interpretation and drafted the manuscript. All authors have read and approved the final manuscript.

## Supplementary Material

Additional file 1: Figure S1 Distribution of all investigated cytokines positive cells by immunocytochemistry analysis. Consecutive tissue sections of case 1 (intratumoral tissues: a, c, e, g, i and k) and case 57 (peritumoral tissues: b, d, f, h, j and l) using immunocytochemistry methods showed different distribution patterns of IL-RA (a and b), IL-17RB (c and d), IL-17RC (e and f), IL-17RD (g and h), IL-17RE (i and j) and IL-17 (k and l), respectively (x 200). Click here for file

Additional file 2: Figure S2 The representative flow cytometry data from 10 haemangioma patients. Click here for file
