# Supplementary figures and images for: High expression of IL-17 and IL-17RE associate with poor prognosis of hepatocellular carcinoma
Source: J Exp Clin Cancer Res. 2013 Jan 11;32(1):3. doi: 10.1186/1756-9966-32-3 (PMC3621615; doi:10.1186/1756-9966-32-3)

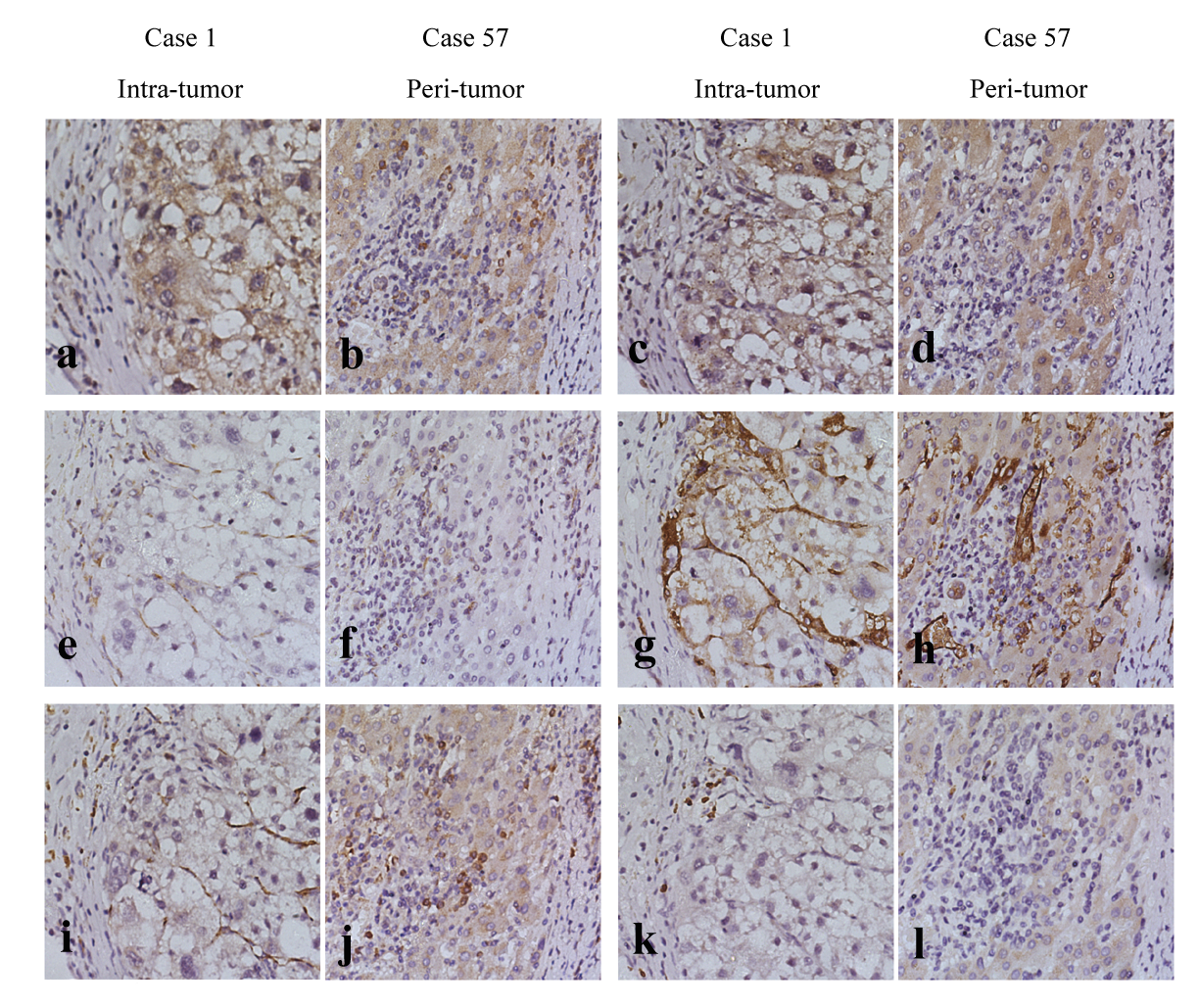

Supplement: Additional file 1: Figure S1 — Distribution of all investigated cytokines positive cells by immunocytochemistry analysis. Consecutive tissue sections of case 1 (intratumoral tissues: a, c, e, g, i and k) and case 57 (peritumoral tissues: b, d, f, h, j and l) using immunocytochemistry methods showed different distribution patterns of IL-RA (a and b), IL-17RB (c and d), IL-17RC (e and f), IL-17RD (g and h), IL-17RE (i and j) and IL-17 (k and l), respectively (x 200). [file 1756-9966-32-3-S1.tiff]

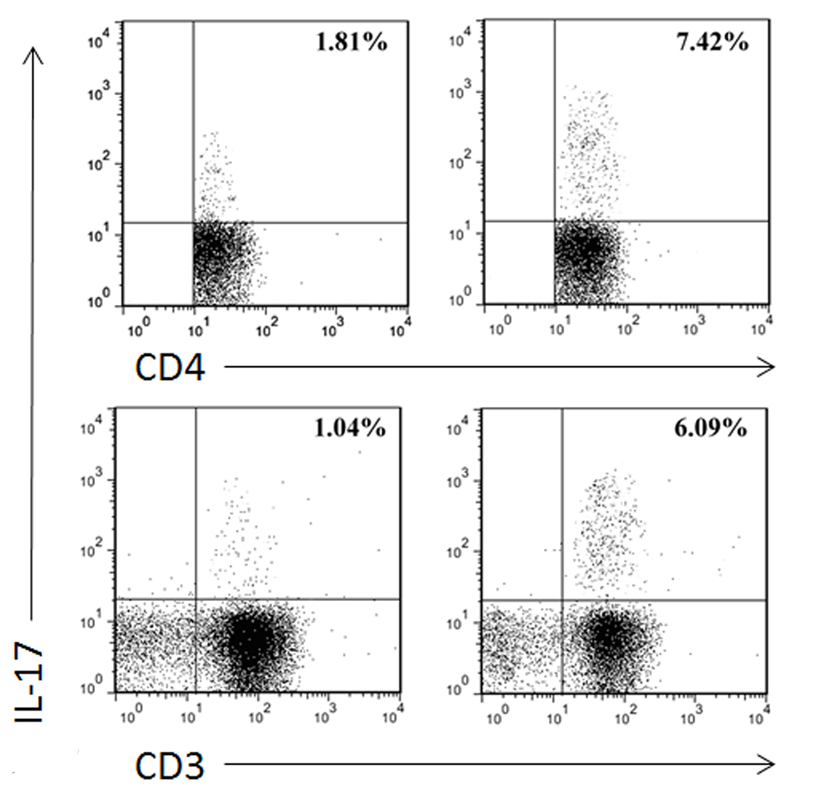

Supplement: Additional file 2: Figure S2 — The representative flow cytometry data from 10 haemangioma patients. [file 1756-9966-32-3-S2.tiff]
